# Size-Sensitive Perceptual Representations Underlie Visual and Haptic Object Recognition

**DOI:** 10.1371/journal.pone.0008009

**Published:** 2009-11-24

**Authors:** Matt Craddock, Rebecca Lawson

**Affiliations:** School of Psychology, University of Liverpool, Liverpool, United Kingdom; University of Leuven, Belgium

## Abstract

A variety of similarities between visual and haptic object recognition suggests that the two modalities may share common representations. However, it is unclear whether such common representations preserve low-level perceptual features or whether transfer between vision and haptics is mediated by high-level, abstract representations. Two experiments used a sequential shape-matching task to examine the effects of size changes on unimodal and crossmodal visual and haptic object recognition. Participants felt or saw 3D plastic models of familiar objects. The two objects presented on a trial were either the same size or different sizes and were the same shape or different but similar shapes. Participants were told to ignore size changes and to match on shape alone. In Experiment 1, size changes on same-shape trials impaired performance similarly for both visual-to-visual and haptic-to-haptic shape matching. In Experiment 2, size changes impaired performance on both visual-to-haptic and haptic-to-visual shape matching and there was no interaction between the cost of size changes and direction of transfer. Together the unimodal and crossmodal matching results suggest that the same, size-specific perceptual representations underlie both visual and haptic object recognition, and indicate that crossmodal memory for objects must be at least partly based on common perceptual representations.

## Introduction

Visual object constancy is the ability to consistently identify objects despite wide variation in their appearance attributable to such causes as a change in orientation between viewing instances [1]. Recent research has started to investigate the attainment of object constancy in haptic object recognition, and how haptic and visual object recognition compare. When compensating for changes of object orientation, similar overall patterns of performance to those observed in vision have been found in the haptic modality [2]–[5]. In Craddock and Lawson [6] we established that there are also similar costs of size changes for visual and haptic familiar object recognition. Here, we extend that research to examine whether size-sensitive representations are modality-specific or are shared across the visual and haptic modalities.

One problem with comparing the effects of variations such as orientation on different modalities is that it is not clear how to match changes across modalities. We will argue that, in contrast to orientation, the effects of size changes may be relatively straightforward to equate across vision and haptics. This means that it is of particular theoretical interest to compare the influence of irrelevant size changes on visual versus haptic object recognition. In the present experiments we used the same method and well-controlled stimuli as Lawson [4] used to examine the effects of orientation changes on unimodal and crossmodal visual and haptic object recognition.

### Similarities between Visual and Haptic Object Recognition

Several lines of evidence have demonstrated striking similarities between visual and haptic object recognition. There is substantial overlap between the neural areas invoked during visual and haptic object recognition [7], particularly in the lateral occipital complex (LOC) [8]–[13] and intraparietal sulcus (IPS) [14]–[15]. Amedi et al. designated the area of overlap between visual and touch recognition in LOC as the lateral occipital tactile-visual (LOtv) area after finding that auditory information relevant to object identity did not elicit activity in this area [8]. They argued that since audition contributes little to the perception of 3D shape, unlike vision and touch, the LOtv is probably involved directly in the recovery of 3D shape. Amedi et al. [16] found that shape information conveyed by a visual-to-auditory sensory substitution device, which converts visual shape information into an auditory stream using a variety of auditory parameters to represent different aspects of the visual image, also activates LOtv. Thus, the LOtv may be driven by geometric shape independent of the sensory input modality [7].

The convergence of activity resulting from visual and haptic object processing at similar neural loci suggests that the two modalities may share representations of shape. Consistent with this, behavioural evidence indicates that there is efficient crossmodal transfer between vision and haptics. Reales and Ballesteros [17] found that unimodal and crossmodal priming for familiar objects was equivalent. However, crossmodal transfer is not always perfect, so there is unlikely to be full perceptual equivalence between the two modalities [18], [19]. For example, Easton, Greene and Srinivas [20] found that although modality changes did not impair priming of 2D patterns or of 3D objects, it did impair performance on an old/new recognition task. Furthermore, Norman, Norman, Clayton, Lianekhammy, and Zielke [21] found that unimodal visual shape matching was better than unimodal haptic shape matching or crossmodal shape matching.

Cooke, Jäkel, Wallraven and Bülthoff [22] conducted a multidimensional scaling analysis of visual and haptic ratings of similarity between pairs of novel objects, and found that the ratings from both modalities were influenced by shape and texture. Vision weighted shape as more important than texture for determining similarity, whereas haptics weighted shape and texture as equally important. Nevertheless, the same perceptual map could account for the pattern of ratings from both modalities, consistent with the hypothesis that the two modalities share common representations.

The evidence that vision and haptics share representations based on geometric shape is compelling, yet the properties of this common perceptual representation, its relationship to unimodal representations, and its broader significance to object recognition are unclear. A key issue for models of object recognition has been to understand how we achieve object constancy by abstracting away from irrelevant variation in the input caused by changes in viewing position and lighting conditions (e.g. [23]–[26]).

The effects of changes of orientation on visual object recognition have been the subject of much empirical research and debate (e.g. [1], [27]–[30]). Generally, the results of these studies and others indicate that visual object recognition is orientation-sensitive (see Peissig & Tarr [31] for a review). Recent behavioural research has found that haptic object recognition is also orientation-sensitive [2]–[5], [32]. All of these studies found broadly similar effects of orientation changes on unimodal visual and haptic object recognition which imply that similar orientation-sensitive representations are used by both modalities. If both modalities use orientation-sensitive representations, then information about orientation might be retained by an object representation which supports recognition across both vision and haptics. The orientation-sensitivity of crossmodal recognition has been tested directly. However, the results, as reviewed below, have been mixed.

Newell et al. [5], using novel objects constructed from LEGO bricks, found that crossmodal visual-to-haptic (VH) and haptic-to-visual (HV) object recognition was orientation-sensitive. However, performance was better when objects were rotated by 180° from study to test than when objects had the same orientation. This was the opposite pattern of orientation-sensitivity than that for unimodal recognition. They suggested that the surface which was perceived determined performance, and that the hands preferentially explored the rear of objects whereas the eye perceived the front of objects. Thus their results suggest that haptics and vision share common, perceptual representations, since performance was always better when the same surfaces were perceived, resulting in opposite directions of orientation-sensitivity between unimodal and crossmodal recognition.

However, Lacey et al. [3] argued that Newell et al.'s results were an artefact of their stimuli. Newell et al.'s stimuli were elongated along their vertical, y-axis and haptic encoding of their near surface was relatively difficult given the biomechanical constraints of the hand. Thus, the ease of acquiring shape information from the near and far surfaces of the stimuli differed. Lacey et al. instead used stimuli which were elongated along their z-axis. Using a similar task to Newell et al., they found that crossmodal recognition was orientation-invariant irrespective of the direction of transfer. Lacey et al. argued that a high-level, spatial object representation underpins crossmodal recognition, and that this representation may be constructed from lower-level, unimodal, orientation-sensitive representations. Using the same stimuli, Lacey et al. [32] trained participants to recognise the objects as accurately when they were rotated as when they were not. They found that this orientation-invariance transferred completely across modalities: Once haptic orientation-invariance had been acquired, visual recognition was also orientation-invariant, and vice versa. They argued that this demonstrated that orientation information is not encoded in the representation underpinning crossmodal recognition.

This conclusion is not consistent with the results reported by Lawson [4], who used the same sequential matching task and the same 3D plastic models of familiar objects as those used in the present article. She found that visual-to-visual (VV), haptic-to-haptic (HH) and VH matching were all orientation-sensitive whereas HV matching was orientation-invariant. The presence of orientation-sensitivity in one direction (VH) but not the other (HV) indicates that crossmodal recognition is not fully orientation-invariant (cf. Lacey et al., [32]), but also that information may not be transferred symmetrically across modalities (cf. Newell et al., [5]).

Thus, while it is clear that there is an object representation accessible to both vision and haptics, it is unclear whether that representation is orientation-sensitive or orientation-invariant: The mixed results above could be attributed to differences in the tasks or stimulus sets employed by the various authors rather than reflecting true differences in the orientation-sensitivity of the object representations. A more interesting possibility is that orientation may not be well matched across the two modalities. There is some evidence consistent with this proposal.

First, in her sequential shape matching task, Lawson [4] manipulated shape discriminability as well as object orientation. She found that for VV matching the cost of ignoring orientation changes increased as the discrimination difficulty increased, whereas for HH and VH matching the cost of ignoring orientation changes was constant irrespective of discrimination difficulty. This suggests that the underlying cause of the orientation-sensitivity observed for VV matching might differ to that for matching involving haptic inputs. Second, Lacey et al. [3] found that the axis of rotation was important for visual but not for haptic object recognition.

Therefore, an important caveat to conclusions drawn from studies which compare haptic and visual orientation-sensitivity is that it is not clear how well-matched changes of orientation are across modalities. The same 90° change in the orientation of an object may be perceived differently in the two modalities, since the mode of exploration differs markedly. For example, from a given viewpoint, vision can only acquire information from the front surface of an object, whereas haptic exploration can encompass most of a small object simultaneously without moving the body. In addition, different frames of reference may be used to encode object orientation visually versus haptically. If orientation is coded using a reference frame based on the sensor (the eye or the hand) then vision and haptics would encode different representations even if the same object was presented to a participant at a fixed position within the environment.

These differences make it hard to interpret patterns of orientation-sensitivity in unimodal and crossmodal visual-haptic experiments. Furthermore, focussing on orientation-sensitivity overlooks other potential sensitivities which may help to characterise the representations shared between vision and haptics. We therefore decided to compare the achievement of object constancy across vision and haptics for a different but commonplace source of input variation: size changes. Different members of a given category often vary widely in size (for example, dogs, and books). In addition, the retinal size of an object is a product not only of the object's physical size but also of its distance from the viewer, which the visual system must also compensate for.

### Effects of Size Changes on Visual and Haptic Object Recognition

There has been substantial research into the effects of size changes on 2D visual object recognition, using line drawings of familiar or novel objects [33]–[35], and greyscale [36], [37] or colour [38] photographs of familiar objects. These studies have shown that 2D visual object recognition is typically impaired by changes in size from study to test on old/new recognition or matching (though not on priming) tasks. In comparison, we are not aware of any studies of the effects of size changes on real, 3D visual object recognition and only our own on the haptic recognition of real, 3D objects [6]. We will discuss this study in detail after briefly noting other haptic object recognition studies which have investigated size effects.

Studies using free- or directed-sorting tasks with 2D planar [39] or 3D cubes and spheres [40] found that size was not a salient dimension for either vision or haptics. Furthermore, Lawson [4] showed that people can recognise small-scale 3D models of familiar objects, indicating that haptics can generalise across unusual sizes.

In Craddock and Lawson [6], we examined the effects of size changes on visual and haptic recognition of familiar 3D objects. In Experiment 1, participants first named a set of real, everyday objects, then performed an old/new recognition task. The task was to respond “old” if an object from a given category had been presented in the first block, ignoring any size or shape changes. Half of the participants performed the experiment visually on photographs of the objects; the other half performed it haptically on the real objects while blindfolded. Size changes were similarly disruptive for both visual and haptic recognition. In Experiment 2, participants performed a haptic sequential shape-matching task on 3D plastic models of familiar objects. Again there was a cost of ignoring irrelevant size changes: performance on match trials (such as when a car was followed by a car) was slower and less accurate when a small car was presented after a large car, or vice versa, than when the same-sized car was presented twice. These two experiments provided the first demonstration of a cost to generalising across size changes in haptics with 3D objects. The first experiment showed that these size change costs occur even when there are size-invariant cues such as texture or temperature available, since the stimuli were real, familiar objects. Furthermore, these size costs were comparable to those observed in vision.

If both vision and haptics use size-sensitive representations, then object representations that can be accessed by either modality may also be size-sensitive. This hypothesis was tested in the present studies. Given that an object's physical size is not contingent upon its spatial relationship to an observer, unlike an object's orientation, then if vision and haptics encode physical size similarly size changes should, in turn, be perceived similarly by both modalities. Furthermore, larger objects take longer to fully explore than smaller objects for both vision and haptics, and, although preferred size may differ, both modalities suffer from a lack of resolution as objects become smaller [38], [41]. As a result it may be more informative to compare the effects of size changes than the effects of orientation changes when contrasting visual to haptic object recognition.

There were important limitations to our previous finding of similar size-sensitivity in visual and haptic object recognition. In Experiment 1 of Craddock and Lawson [6], participants in the visual condition saw only 2D photographs of the familiar 3D objects rather than the actual objects, whereas participants in the haptic condition felt the actual objects. The photographs depicted the objects in a rich and consistent 3D context, and thus provided good information about the absolute size of the objects. This contrasts to most previous studies investigating the effects of size changes on visual object recognition, which have presented 2D images of 3D objects shown in isolation against a blank background without strong cues to their actual physical size or 3D location [33], [36], [38]. Nevertheless, the depth cues available in the visual and haptic conditions were not well matched in this study. Furthermore, there was variation in the direction and magnitude of the size changes used in Experiment 1 because real, everyday objects were presented. Experiment 2 addressed this latter concern by using consistent and counterbalanced size changes, but only tested haptic, not visual matching. Thus, our conclusions about visual versus haptic object recognition from Craddock and Lawson [6] were necessarily limited.

We addressed these issues in two experiments which used a task-irrelevant size transformation to provide evidence about whether the same perceptual representations are used in visual and haptic object recognition. In Experiment 1, we compared unimodal VV matching with unimodal HH matching. Participants performed both VV and HH matching, and the same 3D objects were presented to each modality using the same apparatus, intermingled trials and matched timing. First, this tested whether there is a cost of generalising over visual size changes for 3D objects, an extension of Craddock and Lawson's [6] finding of a size-change cost for 2D photographs of 3D objects. This has not previously been tested. Second, this allowed us to compare unimodal visual and haptic costs of size changes. In Experiment 2, we used the same task and stimuli as in Experiment 1 but participants performed crossmodal VH and HV matching. This provided a more direct test of whether the common representations involved in visual and haptic object recognition are size-sensitive.

## Materials and Methods

### Experiment 1

#### Participants

Twenty-four students from the University of Liverpool participated in return for course credit. Ages ranged from 18 to 57, with most participants aged 18 or 19. Five participants were male, 19 female. Twenty-two participants were right-handed; two were left-handed. Both Experiment 1 and Experiment 2 were approved by the School of Psychology Ethics Committee, University of Liverpool, Liverpool, UK, and written consent was obtained from each participant prior to participation.

#### Stimuli and apparatus

**Figure 1:**
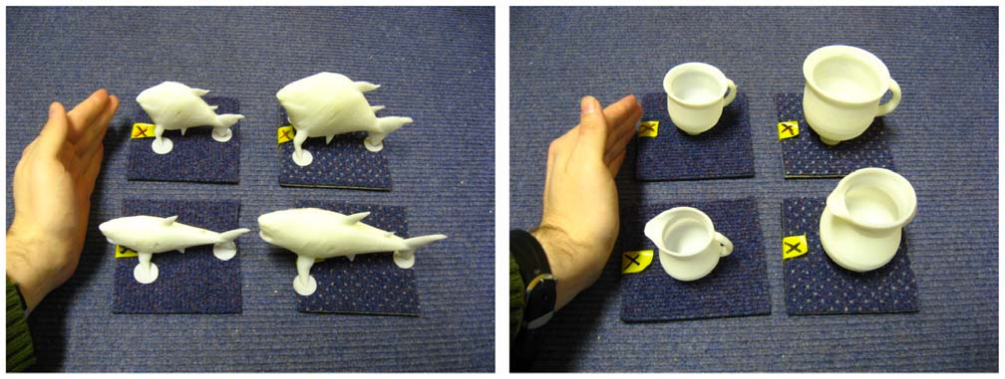
Example morph sets. Examples of two sets (fish-shark and cup-jug) of the stimuli. Each photograph shows the small exemplars on the left and large exemplars on the right.

The stimuli comprised a small and a large version of a startpoint morph and of an endpoint morph for each of 20 familiar object morph sets (see [2] and [4] for further details). The startpoint and endpoint morphs were similarly shaped objects but would normally be given different names, e.g., bath-sink, bed-chair and horse-giraffe (see Table 1). The small version of a given morph was 75% of the width, height and depth (so 42% of the volume) of the large versions. Note that for the majority of objects even the large version was considerably smaller than real life exemplars of the object, since all of the morphs could be comfortably grasped by one hand. All 80 stimuli (two sizes×two morphs×20 morph sets) were 3D white rigid plastic shapes printed using a Dimension 3D ABS-plastic printer, see Figure 1.

**Table 1:** List of familiar object morph pairs.

Banjo-Guitar	Bath-Sink	Bed-Chair	Bench-Chair
Bottle-Watering Can	Camel-Llama	Car-Van	Chair-Stool
Cup-Jug	Dog-Pig	Fish-Shark	Frog-Lizard
Giraffe-Dog	Gun-Spray bottle	Holepunch-Stapler	Horse-Giraffe
Key-Sword	Knife-Spoon	Pencil-Nail	Ship-Submarine

Each morph was glued upright onto the centre of a 10 cm square base made of carpet tile. Yellow tape marked the middle of one side of this base; the object was oriented so that its front was next to the yellow tape. The experimenter positioned objects by placing the base into a 10.5 cm square hole cut into a surround made of a carpet tile. One side of this hole was marked with green tape. The yellow tape at the front of each object was always lined up with the green tape.

The object was hidden from the participant's view by card, a board, and a clouded glass screen. Behind and perpendicular to this glass screen was a 12 cm square aperture through which the participant's right hand entered in order to touch the object on haptic trials or to begin each visual trial. An infra-red beam shone across this slot, placed so that it was broken when the participant's hand entered the slot. When this beam was broken a detector sent a signal to the computer controlling the experiment. Participants responded using a button box placed on the table in front of the glass screen and next to their left hand.

#### Design and procedure

All participants completed one block of 80 trials comprising four sub-blocks of 20 trials. Across the full block of 80 trials there were two match trials and two mismatch trials for each morph set. One of each of these two trials presented both objects at the same size and the other trial presenting the second object at a different size. Both of the two mismatch trials presented the same distractor morph (once as the small and once as the larger version) as the second object. Half of the 80 trials presented both objects visually (VV trials) and half presented both objects haptically (HH). The two trial types were interleaved using an ABBA sequence.

One group of ten morph sets was presented on 40 of the trials in a block. The other group of ten morphs sets was presented on the remaining 40 trials. For half of the participants, the first object presented on a given trial was the startpoint morph (e.g. bath) if it was from the first group of ten morph sets and the endpoint morph (e.g. sink) if it was from the second group of ten morph sets. This assignment was reversed for the remaining participants. On match trials, the second object presented was the same startpoint or endpoint morph as the first object. On mismatches, the second object presented was the startpoint morph if the endpoint morph had been presented first or the endpoint morph if the startpoint morph had been presented first. Note that this design ensured that the matching task was quite difficult, since only objects with related shapes (such as a shark then a fish or a cup then a jug, see Figure 1), were presented on mismatch trials. The order of trials in each sub-block was fixed and an equal number of participants in each condition received the forward and reversed version of this order. Also in each condition, one participant received the trials using the sequence HH-VV-VV-HH, while a second participant received the same sequence of trials using the sequence VV-HH-HH-VV.

The experiment was run on a computer using E-Prime version 1.1 experimental presentation software (Psychology Software Tools Inc., Pittsburgh, PA). At the start of each trial, the experimenter placed the first object into position behind the screen then triggered the computer to play the word “look” on VV trials or the word “touch” on HH trials. This signalled to the participant that they could start to move their right hand through the aperture. The computer recorded when their hand broke the infrared beam across the slot. On VV trials, the screen cleared 500 ms after the beam was broken. This 500 ms delay compensated for the extra time after breaking the beam for participants to move their hand to the object in the HH condition. The screen then clouded 4500 ms after it had cleared. On VV trials they stopped moving their right hand once the beam was broken so their hand did not go near to the object. On HH trials, the screen remained opaque throughout but their right hand could explore the object for five seconds. Five seconds after the beam was broken the words “stop now” were played by the computer, signalling that the participant should withdraw their hand from the slot. The experimenter then removed the first object and either put the same object back behind the screen on match trials or replaced it with a different object on mismatch trials. The experimenter then triggered the computer to play the word “look” or “touch”, and the participant put their hand back through the aperture. In both conditions, the trial concluded when the participant responded, with the screen remaining clear until that time during VV trials and remaining opaque throughout on HH trials.

Participants decided whether the two successively presented objects had the same shape and responded with a speeded keypress. The computer recorded the time from when their right hand broke the infrared beam until they responded with their left hand by pressing one of two buttons (marked “same” and “different”) on a response button box. People were told to ignore any difference in the size of the first and second objects. They were also warned that on mismatches the two objects might have very similar shapes. After they had responded, they heard either a high or a low double tone as feedback which indicated a correct or incorrect response respectively. Participants completed a block of ten practice trials prior to starting the experimental block. These trials were identical to the final ten experimental trials.

After the first object had been presented it was always removed from the apparatus. A second object (the distractor on mismatches and an object from the same morph set as the first object on matches) was then taken from the storage shelf and placed next to the first object. Finally, one of these two objects was put into the apparatus as the second object on a trial. This procedure ensured that participants could not determine whether they were going to be given a match or a mismatch trial from the movements or sounds made by the experimenter. At the end of the study, participants were asked whether they had only used haptic information in the haptic condition to make their responses, or if they had also used auditory or visual information, such as the sounds of the experimenter moving objects or seeing the objects. None reported the use of information other than that gathered by touching or seeing the objects as appropriate.

### Experiment 2

#### Participants

Twenty-four students from the University of Liverpool participated in return for course credit. Ages ranged from 18 to 26. Twenty-two were right-handed, two left-handed. Three were male, 21 female.

#### Design and procedure

Experiment 2 was identical to Experiment 1 except that the two objects on each trial were presented to different modalities. If the first object was presented visually, then the second object was presented haptically and vice versa. Half of the trials presented the first object visually and the second object haptically (VH trials), and half presented the first object haptically and the second object visually (HV trials). Trials were ordered using the same ABBA design as in Experiment 1, with VH trials replacing VV trials and HV trials replacing HH trials.

## Results

### Experiment 1

In Experiment 1, participants performed a sequential shape matching task using plastic, 3D models of familiar objects. The models were scaled to be approximately hand-sized, and were all made from the same, rigid plastic. Thus, all the models had the same surface texture, temperature and compliance. Furthermore, the weight of the models bore little relation to the weight of the real exemplars of the modelled object category. Thus, while participants could use normal haptic exploratory procedures [42], [43], there were no non-shape cues to identity. The absence of non-shape cues should maximize the influence of our primary manipulation, changes in size, on participants' performance.

Participants studied an object for 5 seconds. They were then presented with either the same shaped object on match trials or a different shaped object on mismatch trials. On both match and mismatch trials, the first and second objects were the same size on half of the trials and were different sizes on the remaining trials. The task was to detect shape changes and ignore size changes. Both objects on a trial were presented to the same modality (i.e. trials were visual-to-visual, VV, or haptic-to-haptic, HH). Participants were informed about the modality of each upcoming trial using a verbal cue (“touch” or “look”). Based on the results of Craddock and Lawson [6], we expected size changes to disrupt VV and HH matching about equally.

#### Analysis

Repeated-measures analyses of variance (ANOVA) were conducted on mean correct reaction times (RTs) and mean percentage errors for matches and mismatches separately. On matches, same-shape responses were correct. On mismatches, different-shape responses were correct. Reaction times shorter than 350 ms or longer than 5000 ms on VV trials and shorter than 750 ms or longer than 10000 ms on HH trials were discarded as outliers (less than 1% of trials). No participants were replaced. Size (same or different) and modality (VV or HH) were used as within-participants variables. Subscripts *F*_p_ and *F*_i_ denote by-participants and by-items analyses *F*-values respectively. There was no indication of a speed-accuracy trade-off in the results of Experiment 1, since longer RTs were not associated with fewer errors.

#### Same-shape matches

Size was significant for RTs [*F*_p_(1,23) = 28.004, *p*<.001; *F*_i_(1,19) = 48.234, *p*<.001] and errors [*F*_p_(1,23) = 22.821, *p*<.001; *F*_i_(1,19) = 36.782, *p*<.001]. Matching on same-size trials (1945 ms; 3% errors) was 210 ms faster and 11% more accurate than matching on different-size trials (2155 ms; 14% errors). There was therefore a substantial cost of generalising over size changes on both the speed and accuracy of performance.

Modality was significant for RTs [*F*_p_(1,23) = 450.292, *p*<.001; *F*_i_(1,19) = 986.128, *p*<.001] and errors [*F*_p_(1,23) = 22.594, *p*<.001; *F*_i_(1,19) = 7.472, *p* = .013]. VV matching (1163 ms; 5% errors) was 1774 ms faster and 7% more accurate than HH matching (2937 ms; 12% errors).

**Figure 2:**
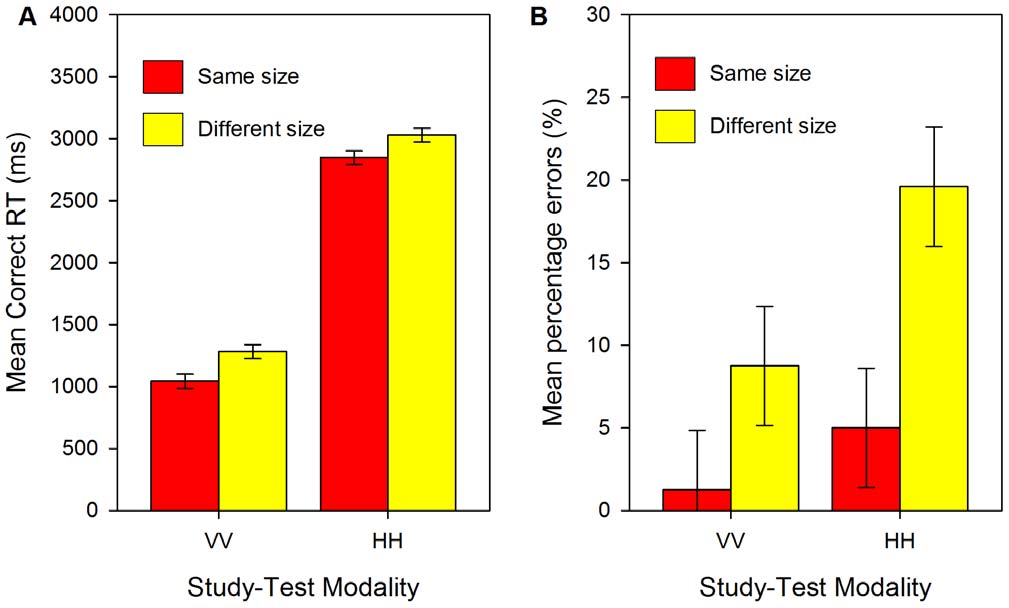
Results of Experiment 1: Unimodal matching. (a) Mean correct RTs (ms) and (b) mean percentage errors for unimodal, visual-to-visual (VV) and haptic-to-haptic (HH) matches in Experiment 1. Error bars show 95% within-participant confidence intervals calculated using the error term of the modality × size interaction (see [48], [49]).

There was no size × modality interaction for RTs [*F*_p_(1,23) = 1.043, *p* = .3, see Figure 2a; *F*_i_(1, 19) = .666, *p* = .4], but there was a marginal interaction for errors [*F*_p_(1,23) = 4.136, *p* = .05, see Figure 2b; *F*_i_(1,19) = 4.125, *p* = .06]. On VV trials, same-size matching was 238 ms faster and 8% more accurate. On HH trials, same-size matching was 183 ms faster and 15% more accurate.

#### Different-shape mismatches

Mismatch trials were not the focus of this study since they presented two different shaped objects (e.g., frog then lizard). This shape change often produced a substantial size change in at least one dimension (for example, the lizard was much longer than the frog). It is therefore difficult to interpret the results of mismatches in terms of the effects of the size-change manipulations. Nevertheless, the mismatch results are presented here for completeness.

Size was not significant for RTs [*F*_p_(1,23) = .008, *p* = .9; *F*_i_(1,19) = .436, *p* = .5] or errors [*F*_p_(1,23) = .008, *p* = .9; *F*_i_(1,19) = .014, *p* = .9]. Modality was significant for RTs [*F*_p_(1,23) = 267.690, *p*<.001; *F*_i_(1,19) = 641.978, *p*<.001] and errors [*F*_p_(1,23) = 35.276, *p*<.001; *F*_i_(1,19) = 19.301, *p*<.001]. VV mismatches (1162 ms; 4% errors) were 1868 ms faster and 19% more accurate than HH mismatches (3030 ms; 23% errors). There was no size × modality interaction for RTs [*F*_p_(1,23) = .358, *p* = .6; *F*_i_(1,19) = .013, *p* = .9] or errors [*F*_p_(1,23) = .015, *p* = .9; *F*_i_(1,19) = .041, *p* = .8].

#### Discussion of experiment 1

The results were clear: for both vision and haptics, sequential shape matching was performed faster and more accurately when a given object was presented both times at the same size compared to when it changed size from the first to the second presentation.

These results are the first demonstration of size change costs to visual recognition using 3D objects. The majority of previous research investigating visual size change effects presented photographs or line drawings of objects set against blank backgrounds with no environmental context. Without an environmental context, size changes could either be interpreted as changes of distance or as attributable to rescaling of an image. We previously demonstrated that size change effects occur even when the photographs show objects within a standard scene which provided good information about physical object size [6]. The current study extended this result by presenting 3D objects with full, consistent cues to actual size and presented at a fixed distance. Here, differences in size would have been seen as changes in the physical size of an object and yet size change costs were still observed.

One reason for visual size-sensitivity could be that the reaching movement made by participants invoked action representations associated with reaching and grasping (e.g. [44], [45]), and that these representations might contain size-specific information. However, the activation of action representations seems unlikely to have had an important influence on our results for a number of reasons. First, such representations are also invoked without reaching [44], and may reflect more general, less exemplar-specific information such as motion of action (e.g. pour versus twist) [44] or type of grip (e.g. power versus precision) [46]. Second, our stimuli were scale models of familiar objects, so many of their associated action representations would not be appropriate. For example, one cannot sit on a hand-sized model of a stool. Third, mismatches presented shapes which would generally invoke similar actions (e.g. chair-stool; stapler-hole puncher). Thus, size-specificity due to action representations should also have induced size-specific performance on mismatches, which we did not observe. Third, many of our models were of animals, which do not have clearly associated action representations. Finally, participants stopped their movement as soon as the object was visible, and thus the reaching movement made on visual trials was a simple, stereotyped motion aimed simply at triggering the apparatus and not at grasping the object.

There was also a substantial cost of size changes for haptic recognition. It was therefore clear that both vision and haptics used size-sensitive representations of shape to perform the task. Experiment 1 used the same task, the same apparatus and a within-participant manipulation of modality and the cost on RTs of compensating for size changes was similar for visual and haptic recognition. This finding is consistent with the claim that, notwithstanding the differences between initial sensory processing across the two modalities, subsequent stages of perceptual object processing are similar for vision and touch.

Contrary to the predictions of this claim, there was a marginal interaction between size change and modality for errors, indicating that the absolute size change cost was somewhat smaller for vision than for haptics. However, as Figures 2a and 2b show, VV matching was also much faster and more accurate overall than HH matching. Our analysis of absolute costs may therefore have underestimated the size cost for VV matching. In contrast, when comparing relative costs, VV size changes increased RTs by 23% and errors by 600%, whilst HH size changes increased RTs by 6% and errors by 292%. There may also have been a ceiling effect for errors in the same-size VV condition, see Figure 2b.

These differences in baseline performance across the modalities are an inevitable consequence of the fundamental differences between normal processing by vision and haptics, such as the rate and means of acquisition of shape information. Overall levels of performance can usually only be equated across the modalities by artificially constraining information acquisition, for example by restricting vision to a narrow field of view [47]. An alternative approach was used in Experiment 2: Crossmodal matching was investigated. If size-sensitivity is weaker for visually compared to haptically encoded representations then there should be a reduced cost for VH size changes than for HV size changes.

Importantly, testing crossmodal as well as unimodal matching permits a comparison of size-sensitivity across trials with similar baseline performance, since the modality to which the second object presented is the main determinant of overall performance. Specifically, VV and HV performance are similarly fast whereas HH and VH performance are similarly slow (e.g. [4]). A cross-experiment analysis is presented below, after the results of Experiment 2 have been reported.

### Experiment 2

The results of Experiment 1 suggested that the cost of size changes was similar for VV and HH matching, consistent with an account of object recognition in which vision and haptics share the same or similar perceptual representations. These results are also similar to those observed by Lawson [6] for VV and HH matching across orientation changes using the same task, stimuli and apparatus. However, as Lawson [6] demonstrated, this superficial similarity needs to be investigated further since important differences in orientation-sensitivity have also been observed between the two modalities for crossmodal matching and when another factor, shape discriminability, is manipulated. Therefore, in Experiment 2 we used the same sequential shape matching task as in Experiment 1 to test crossmodal visual-to-haptic (VH) and haptic-to-visual (HV) matching.

The results of Experiment 1 also suggested that both visual and haptic encoding produces size-sensitive representations. Any representation mediating crossmodal recognition may therefore also be size-sensitive. Alternatively, if crossmodal matching is mediated by a more abstract shape representation, or a spatial representation which does not encode size, then there should be no cost of size changes to crossmodal shape matching. Furthermore, any difference in the size-sensitivity of representations encoded visually versus haptically should modulate size change costs according to the direction of transfer. If visual representations are less size-sensitive than haptic representations, the cost of size changes should be reduced for VH compared to HV matching.

#### Analysis

Repeated-measures ANOVAs were conducted on mean correct reaction times and mean percentage errors for matches and mismatches separately. On matches, same-shape responses were correct. On mismatches, different-shape responses were correct. Reaction times (RTs) shorter than 350 ms or longer than 5000 ms on HV trials and shorter than 750 ms or longer than 10000 ms on VH trials were discarded as outliers (less than 1% of trials). Three participants were replaced as they made errors on over 30% of trials. Size (same or different) and modality (VH or HV) were used as within-participants variables. As in Experiment 1, there was no indication of a speed-accuracy trade-off in the results of Experiment 2, since longer RTs were not associated with fewer errors.

#### Same-shape matches

Size was significant for RTs [*F*_p_(1,23) = 12.334, *p* = .002; *F*_i_(1,19) = 26.922, *p*<.001] and for errors [*F*_p_(1,23) = 17.040, *p*<.001; *F*_i_(1,19) = 40.619, *p*<.001]. Same-size matches (2464 ms; 9% errors) were 243 ms faster and 11% more accurate than different-size matches (2707 ms; 20% errors). There was a substantial cost of generalising over size changes on both the speed and accuracy of performance.

Modality was significant for RTs [*F*_p_(1,23) = 247.283, *p*<.001; *F*_i_(1,19) = 377.671, *p*<.001] and for errors [*F*_p_(1,23) = 8.144, *p* = .009; *F*_i_(1,19) = 7.715, *p* = .01]. HV matching (1535 ms; 18% errors) was 2100 ms faster but 8% less accurate than VH matching (3635 ms; 10%).

**Figure 3:**
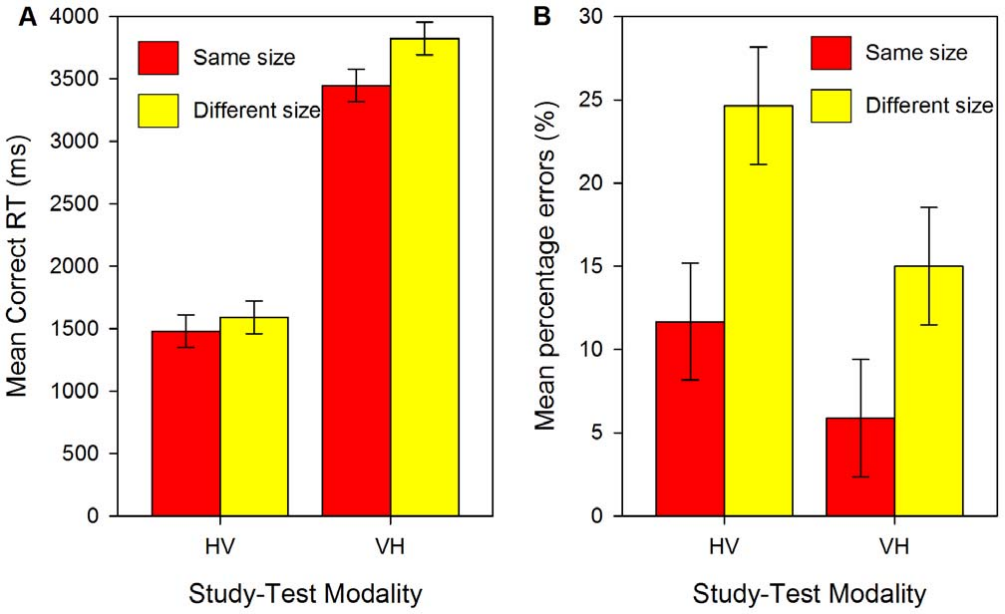
Results of Experiment 2: Crossmodal matching. (a) Mean correct RTs (ms) and (b) mean percentage errors (%) for crossmodal, haptic-to-visual (HV) and visual-to-haptic (VH) matches in Experiment 2. Error bars show 95% within-participant confidence intervals calculated using the error term of the modality × size interaction [48], [49].

The size × modality interaction was significant for RTs [*F*_p_(1,23) = 4.484, *p* = .05, see Figure 3a; *F*_i_(1,19) = 6.591, *p* = .02] but not for errors [*F*_p_(1,23) = 1.275, *p* = .3, see Figure 3b; *F*_i_(1,19) = .941, *p* = .3]. On HV trials, same-size matches were 109 ms faster and 13% more accurate than different-size matches. On VH trials, same-size matches were 377 ms faster and 9% more accurate than different-size matches.

#### Different-shape mismatches

As in Experiment 1, it is difficult to interpret performance on mismatch trials since the shape changes also often produced substantial size changes. Nevertheless, as before, the results are presented here for completeness. There was a weak trend of size for RTs [*F*_p_(1,23) = 3.506, *p* = .07; *F*_i_(1,19) = 3.070, *p* = .1] and for errors [*F*_p_(1,23) = 3.036, *p* = .1; *F*_i_(1,19) = 1.423, *p* = .2]. Same-size mismatches (2703 ms; 23% errors) were 148 ms slower and 4% less accurate than different-size mismatches (2591 ms; 19% errors). Modality was significant for RTs [*F*_p_(1,23) = 300.566, *p*<.001; *F*_i_(1,19) = 240.682, *p*<.001] but not errors [*F*_p_(1,23) = 1.324, *p* = .3; *F*_i_(1,19) = 1.929, *p* = .2]. HV mismatches (1580 ms; 22% errors) were 2134 ms faster than VH mismatches (3714 ms; 25% errors). There was no size × modality interaction for RTs [*F*_p_(1,23) = .784, *p* = .4; *F*_i_(1,19) = 2.713, *p* = .1] or errors [*F*_p_(1,23) = .395, *p* = .5; *F*_i_(1,19) = .503, *p* = .5].

#### Discussion of experiment 2

For both HV and VH crossmodal matches, there was a cost of ignoring irrelevant size changes. This extended the results of Experiment 1 which found size change costs for both VV and HH unimodal matches. The results indicate that crossmodal object recognition depends at least partly on size-specific, perceptual representations rather than solely on more abstract shape representations.

There was no interaction between transfer direction (VH or HV) and the cost of size changes for errors, consistent with the hypothesis that similar object representations were accessed in both cases. However, for reaction times the size cost was larger for VH compared to HV matching. Importantly, though, this difference suggests that now visually-encoded representations were more size-sensitive than haptically-encoded representations, so this effect was in the opposite direction to that found in Experiment 1. This in turn suggests that the reason for the variation in size-sensitivity in both studies is that size-sensitivity is greater when overall responses are slower due to the second object being presented haptically, on VH and HH trials, compared to when the second object is presented visually, on HV and VV trials. Size changes increased RTs by 11% in VH matching and 7% in HV matching, so the relative increase in RTs was similar in both cases.

#### Comparing size change costs for unimodal and crossmodal matching

Since the size change cost was the main measure of interest, we subtracted the RTs and errors for same-size trials from RTs and errors for different-size trials for all conditions. We then performed a mixed ANOVA on this mean size change cost for RTs and errors using second object modality (visual for VV and HV matches or haptic for HH and VH matches) as a within-participants factor and transfer (unimodal for VV and HH matches or crossmodal for HV and VH matches) as a between-participants factor.

There was a non-significant trend of second object modality for RTs [*F*_p_(1,46) = 2.174, *p* = .1; *F*_i_(1,19) = 2.241, *p* = .2], with smaller costs (179 ms) on VV and HV trials than on HH and VH trials (281 ms), but no effect for errors [*F*_p_(1,46) = .443, *p* = .5; *F*_i_(1,19) = .313, *p* = .6].

There was no effect of transfer for either RTs [*F*_p_(1,46) = .108, *p* = .7; *F*_i_(1,19) = .044, *p* = .8] or errors [*F*_p_(1,46) = .000, *p* = 1; *F*_i_(1,19) = .008, *p* = .9].

**Figure 4:**
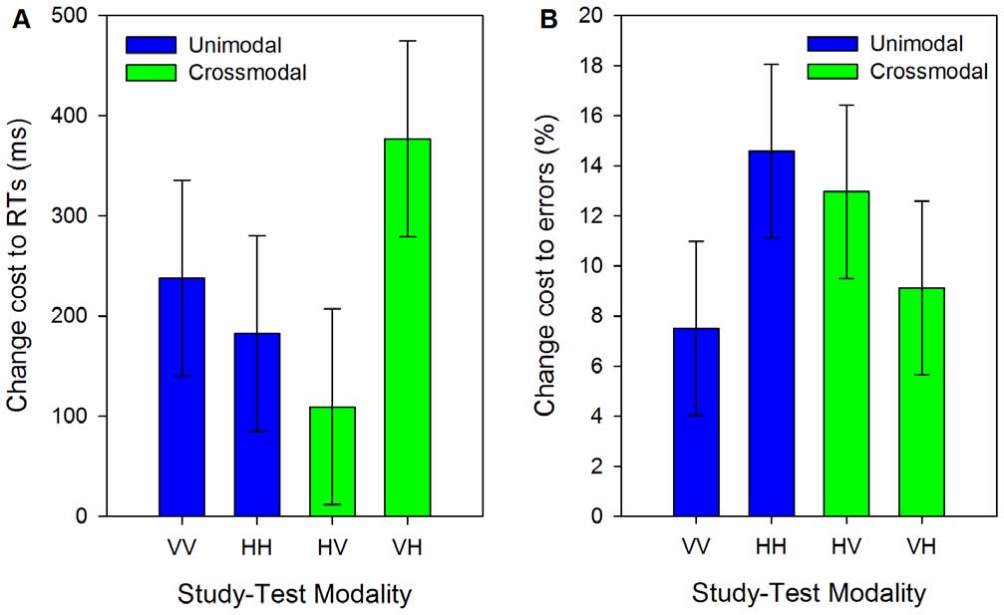
Cross-experiment size-change cost analysis. Size-change cost to (a) mean correct RTs (ms) and (b) mean percentage errors (%) in Experiment 1 (VV and HH matching) and Experiment 2 (HV and VH matching). Error bars show 95% within-participant confidence intervals calculated using the error term of the second object modality × transfer interaction [48], [49].

There was an interaction between second object modality and transfer for both RTs [*F*_p_(1,46) = 5.732, *p* = .02, see Figure 4a; *F*_i_(1,19) = 7.524, *p* = .01] and errors, though only marginally by-items [*F*_p_(1,46) = 5.035, *p* = .03, see Figure 4b; *F*_i_(1,19) = 3.712, *p* = .07]. We conducted post-hoc Tukey's HSD tests on these interactions. For RTs, there was a greater size cost to VH matching than to HH or HV matching. For errors, the size cost was greater for HH matching than for VV matching. No other comparisons were significant.

#### Comparing overall performance for unimodal and crossmodal matching

The above analysis of size change costs examined only the effects of transfer on the size change costs, and thus did not test for a cost of crossmodal transfer per se. Therefore, we compared the results of Experiment 1 and Experiment 2 directly using the same factors as in the separate analyses of those experiments – modality (vision or haptics) and size (same or different) – but with the addition of transfer (unimodal – i.e., Experiment 1 – or crossmodal – i.e., Experiment 2) as a between-participants factor. Only the main effect of transfer is reported here, since the above analyses already report all higher-order interactions of theoretical interest. Crossmodal matching (2585 ms, 14% errors) was 535 ms slower [*F*_p_(1,46) = 18.099, *p*<.001; *F*_i_(1,19) = 182.349, *p*<.001] and 5% less accurate [*F*_p_(1,46) = 9.249, *p* = .004; *F*_i_(1,19) = 7.730, *p* = .012] than unimodal matching (2050 ms, 9% errors).

#### Discussion of cross-experiment analyses

We compared unimodal to crossmodal matching directly by analysing the results of Experiments 1 and 2 together. This revealed a modest decrease in speed and accuracy for crossmodal matching, consistent with previous findings of a cost of transfer across modalities (e.g. [3], [21]).

The analysis of size change costs revealed an interaction between second object modality and transfer. This interaction might be taken as evidence against the hypothesis that the same perceptual representations are involved in visual and haptic object processing. However, the larger size cost on errors for HH compared to VV matches is likely due to differences in overall accuracy across these two conditions, with fewer errors made on VV matches, see Figure 2. Similarly, the larger size costs to RTs for VH than for HV or HH matching may at least in part be due to this condition being the slowest overall. Furthermore, this condition did not produce the largest size costs for errors, see Figure 4b. The modest differences in size change costs across the four conditions appear to mainly reflect variation in overall levels of performance rather than the effects of modality per se. It is also important to note that there were significant size costs in all conditions, and there were no differences between size costs in the unimodal and crossmodal conditions.

## Discussion

Together the two studies reported here tested unimodal (HH and VV) and crossmodal (HV and VH) sequential matching of 3D models of familiar objects. In all four conditions performance was better on same-size relative to size-change matches, indicating that the perceptual shape representations underlying visual and haptic object recognition are size-sensitive. These results extend our previous findings of size-sensitivity in 2D visual and 3D haptic object recognition [6].

The size costs found for VV matches are consistent with previous findings of effects of size changes on 2D images [6], [33]–[38] and extend them to an ecologically important situation in which participants saw real 3D objects in a real 3D environmental context with full depth cues. There were similar size costs for HH matches, providing evidence that the same representations are involved in visual and haptic object recognition.

However, research investigating the effects of orientation transformations on visual versus haptic object recognition has shown that superficial similarities in unimodal performance across the two modalities may be misleading. More fine-grained investigation may reveal important differences between the modalities. For example, Lawson [4] found that an additional factor, discrimination difficulty, had different effects on visual versus haptic matching and crossmodal transfer was orientation-sensitive from vision to haptics but orientation-invariant from haptics to vision. Furthermore, VH and HV crossmodal transfer has also been reported to be orientation-sensitive in both directions [5] and orientation-invariant in both directions [3]. However, note that Newell et al.'s results may not generalise beyond the particular stimuli and orientations that they used [3] whilst in both crossmodal conditions in Lacey et al. [3] there was a trend towards a same-orientation advantage to recognition. Thus, their finding of orientation-invariance may have been due to a lack of statistical power.

Given the difficulty in interpreting these varying results for crossmodal recognition, the present findings provide important evidence about the achievement of object constancy for haptics versus vision by manipulating size rather than orientation changes. Lawson [4] investigated crossmodal matching using the same task, stimuli and apparatus as in the present studies. Experiment 2 here was motivated by her finding of asymmetrical crossmodal transfer effects on orientation sensitivity for VH compared to HV matching. Despite the similarity between these two studies, a different pattern of results was found to that observed by Lawson [4], with size change costs observed for both VH and HV matches.

Our results confirm that both visual and haptic object recognition employ size-sensitive representations, and indicate that each can efficiently access size-specific representations encoded by the other modality. These object representations preserve task-irrelevant perceptual information about a specific encounter with a given object, so are not fully abstract representations of shape or semantic or verbal representations. We suggest that the variation in results for the achievement of object constancy across previous studies may be due to the difficulty in equating object transformations such as orientation across vision and haptics. This difficulty arises from the fundamental differences between the modalities, for example in the amount of the surface of an object that can be explored simultaneously or from a given position and because vision and haptics may encode objects using different frames of reference. Relative to orientation changes, we propose that size transformations provide an important alternative - and arguably superior - means of comparing visual to haptic object recognition.
